# Dataset on open/blind hole-hole interaction in barely visible impact damaged composite laminates

**DOI:** 10.1016/j.dib.2020.106607

**Published:** 2020-12-01

**Authors:** W.L. Lai, H. Saeedipour, K.L. Goh

**Affiliations:** a Newcastle Research & Innovation Institute (NewRIIS), 80 Jurong East Street 21, #05-04, Singapore 609607, Singapore; b Faculty of Science, Agriculture and Engineering, Newcastle University, Newcastle Upon Tyne NE1 7RU, UK; c Republic Polytechnic, School of Engineering, Singapore

**Keywords:** Composite material, Impact damaged, In-plane compression, Infrared thermography, Hole-hole interaction, Hole orientation, Damaged structure

## Abstract

This dataset contains the mechanical properties and structural characteristics with images of the carbon fibre reinforced epoxy composite (CFRP) laminates with open/blind holes. The mechanical dataset are the fracture strength, strain at fracture, strain energy density for resilience, strain energy density to fracture and stiffness of the CFRP laminates for different setups (namely 1 hole, 2 holes parallel to applied load, and 2 holes normal to applied load) from pristine and barely visible impact damage (BVID) specimens, determine from in-plane compression test. The structural-related dataset include thermographs, images of BVID specimens, drilling-induced damage BVID specimens and video clips of crack propagation during in-plane compression testing.

## Specifications Table

**Table:** 

Subject	*Engineering, Composites, Mechanics of materials, Mechanical Engineering*
Specific subject area	Hole application for composite repair, strength of materials, hole-hole interaction and orientation
Type of data	Table, Image, Chart, Graph, Figure, Video
How data were acquired	*Infrared thermography (C-CheckIR, Automation Technology GmbH), Digital camera (Nikon D7200), Mechanical tester (MTS Landmark)*
Data format	*Raw, Analyzed, Filtered*
Parameters for data collection	*Mechanical condition: Pristine, BVID CFRP laminates (24 ply, 100* *mm by 160* *mm)* *Hole setup: (i) no holes, (ii) 1 hole, (iii) 2 holes parallel to load, (iv) 2 holes normal to load* *Hole type: blind, open*
Description of data collection	*Quasi-static indentation (QSI) was performed to induce damaged (*i.e. *BVID) to the samples. Holes were created in the BVID samples by mechanical drilling.* *Imaging and quantifying of the structural data, namely BVID diameter and drilling-induced damage diameter, were acquired using Infrared thermography. Acquisition of load and displacement during in-plane compression test was used to determine fracture strength, strain at fracture, strain energy density for resilience, strain energy density to fracture and stiffness of the CFRP laminates. Imaging and videoing of the crack propagation during in-plane compression test were obtained using digital camera.*
Data source location	*Newcastle University in Singapore/Newcastle Research & Innovation* *Institute Singapore* *Republic Polytechnic, Singapore*
Data accessibility	*Data are available with this article*

## Value of the Data

The data is valuable because it contains important information concerning how hole type, hole-hole interaction and orientation of holes within the damage area affects the mechanical properties of the composite laminate.

The data could be valuable for informing materials engineers about (1) the damaged material structure integrity following the drilling of holes, (2) effects of the hole-hole interaction and the orientation of holes on the mechanical behaviour of the BVID specimen, and (3) performance of different type of holes, namely blind and open holes.

The data could direct future research relating to the optimization of the design of resin-injection method for repairing damage structures [1], [2], as well as new approaches relating to the response of drilling holes in composite materials.

## 1 Data Description

The data consists of the mechanical properties and structural information of the carbon fibre epoxy composite (CFRP) laminate containing different hole types (blind holes and open holes) and hole setups (1 hole, 2 holes parallel to the load, and 2 holes normal to the load) in pristine and barely visible impact damaged (BVID) conditions.

The mechanical data comprises of the fracture strength (*σ_U_*), strain at fracture (*ε_U_*), strain energy density for resilience (*u_E_*), strain energy density to fracture (*u_F_*) and stiffness (E) of the CFRP laminates derived from the stress-strain curves of the specimens subjected to in-plane compression testing to fracture. (Table 1) shows the derived data of the mechanical properties of CFRP laminates with the presence of blind holes. Table 2 shows the derived data of the mechanical properties of CFRP laminates with the presence of open holes. The complete mechanical data can be found in the “Mech_Properties” spreadsheet in the supplementary excel file (Data_Mech_Struct). Of note, the data related to the CFRP laminates without any holes can be retrieved from another data article [3].

**Table 1:** Mechanical properties of CFRP laminates with different blind hole orientations in pristine and damaged state.

	Pristine					Damaged				
	*σ_U_*	*ε_U_*	*u_Y_*	*u_F_*	E	*σ_U_*	*ε_U_*	*u_E_*	*u_F_*	E
	MPa		MJ/m^3^	MJ/m^3^	GPa	MPa		MJ/m^3^	MJ/m^3^	GPa
**1 hole**	309.4	0.0112	0.75	1.75	29.82	186.4	0.0095	0.15	0.76	23.64
**(1H)**	272.7	0.0114	0.49	1.67	29.26	176.9	0.0099	0.14	0.77	20.31
	314.3	0.0121	0.56	1.75	29.92	176.5	0.0099	0.14	0.76	21.25
**2 holes parallel to load**	304.4	0.0116	0.62	1.72	29.71	176.2	0.0097	0.15	0.75	22.38
**(2Hp)**	292.7	0.0114	0.61	1.54	29.45	172.0	0.0092	0.17	0.73	22.63
	317.9	0.0118	0.64	2.04	30.06	171.6	0.0095	0.14	0.70	21.48
	286.9	0.0112	0.59	1.48	30.06	165.2	0.0089	0.16	0.66	22.53
	297.3	0.0121	0.52	1.74	29.13	165.0	0.0090	0.15	0.66	21.89
	307.8	0.0119	0.56	1.64	30.05	177.1	0.0088	0.17	0.69	24.84
**2 holes normal to load**	309.9	0.0118	0.64	1.80	31.15	194.0	0.0105	0.13	0.88	21.58
**(2Hn)**	280.2	0.0113	0.64	1.67	28.27	165.5	0.0089	0.15	0.65	22.56
	262.3	0.0111	0.53	1.35	28.03	183.0	0.0098	0.16	0.79	22.05
	289.8	0.0104	0.66	1.54	32.61	164.5	0.0074	0.26	0.61	24.13
	284.5	0.0106	0.61	1.40	30.74	151.8	0.0080	0.15	0.53	24.22
	293.1	0.0115	0.59	1.65	31.62	162.6	0.0082	0.17	0.58	24.34

*σ_U_* - compressive strength, *ε_U_* - compressive strain at rupture, *u_E_* - strain energy density for resilience, *u_F_* - strain energy density to rupture, E - stiffness.

**Table 2:** Mechanical properties of CFRP laminates with different open hole orientations in pristine and damaged state.

	Pristine state					Damaged state				
	*σ_U_*	*ε_U_*	*u_E_*	*u_F_*	E	*σ_U_*	*ε_U_*	*u_E_*	*u_F_*	E
	MPa		MPa	MPa	GPa	MPa		MPa	MPa	GPa
**1 hole**	291.2	0.0114	0.21	1.72	29.26	181.3	0.0084	0.19	0.77	26.82
**(1H)**	288.7	0.0118	0.19	1.67	29.60	176.1	0.0087	0.17	0.81	24.90
	265.6	0.0111	0.18	1.36	28.50	178.0	0.0090	0.16	0.74	24.62
**2 holes parallel to load**	294.6	0.0110	0.24	1.73	29.95	196.8	0.0102	0.12	0.84	29.38
**(2Hp)**	290.3	0.0117	0.19	1.49	28.94	175.7	0.0092	0.14	0.67	23.12
	325.4	0.0124	0.21	1.79	29.68	190.8	0.0097	0.16	0.82	23.63
	290.7	0.0118	0.22	1.63	28.57	159.2	0.0090	0.15	0.64	24.24
	286.5	0.0117	0.21	1.67	28.47	163.5	0.0091	0.15	0.66	21.90
	306.0	0.0123	0.17	1.72	29.95	166.6	0.0098	0.11	0.68	22.15
**2 holes normal to load**	280.4	0.0126	0.20	1.73	26.16	185.9	0.0105	0.13	0.84	20.67
**(2Hn)**	284.6	0.0127	0.19	1.83	26.40	162.9	0.0089	0.15	0.64	22.21
	291.7	0.0135	0.18	2.12	25.68	176.7	0.0098	0.15	0.77	21.30
	282.2	0.0126	0.17	1.95	25.16	155.6	0.0089	0.14	0.60	21.65
	289.6	0.0122	0.20	1.62	27.01	173.4	0.0090	0.18	0.72	23.23
	287.9	0.0124	0.22	1.76	26.71	166.0	0.0098	0.12	0.68	19.92

*σ_U_* - compressive strength, *ε_U_* - compressive strain at rupture, *u_E_* - strain energy density for resilience, *u_F_* - strain energy density to rupture, E - stiffness.

The structural data comprises images of BVID laminates and drilling-induced damage around holes in BVID laminates taken using infrared thermography, and the following quantitative data:

• diameter of the BVID,
• diameter of the holes,
• drilling-induced damage diameter around the holes,

were derived from the images.

**Fig. 1:**
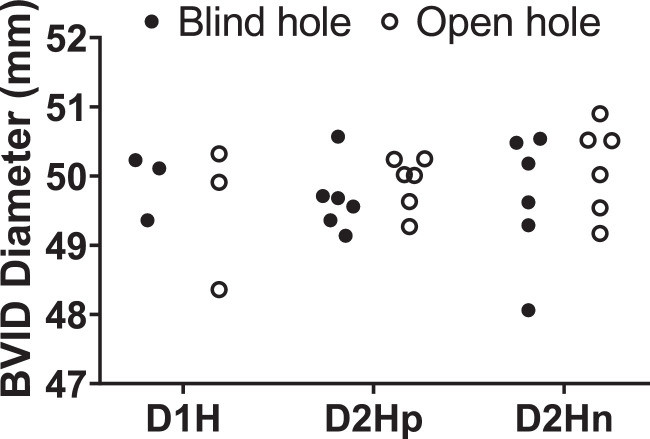
Dot plot of the BVID diameter versus the blind and open hole setup.

**Fig. 2:**
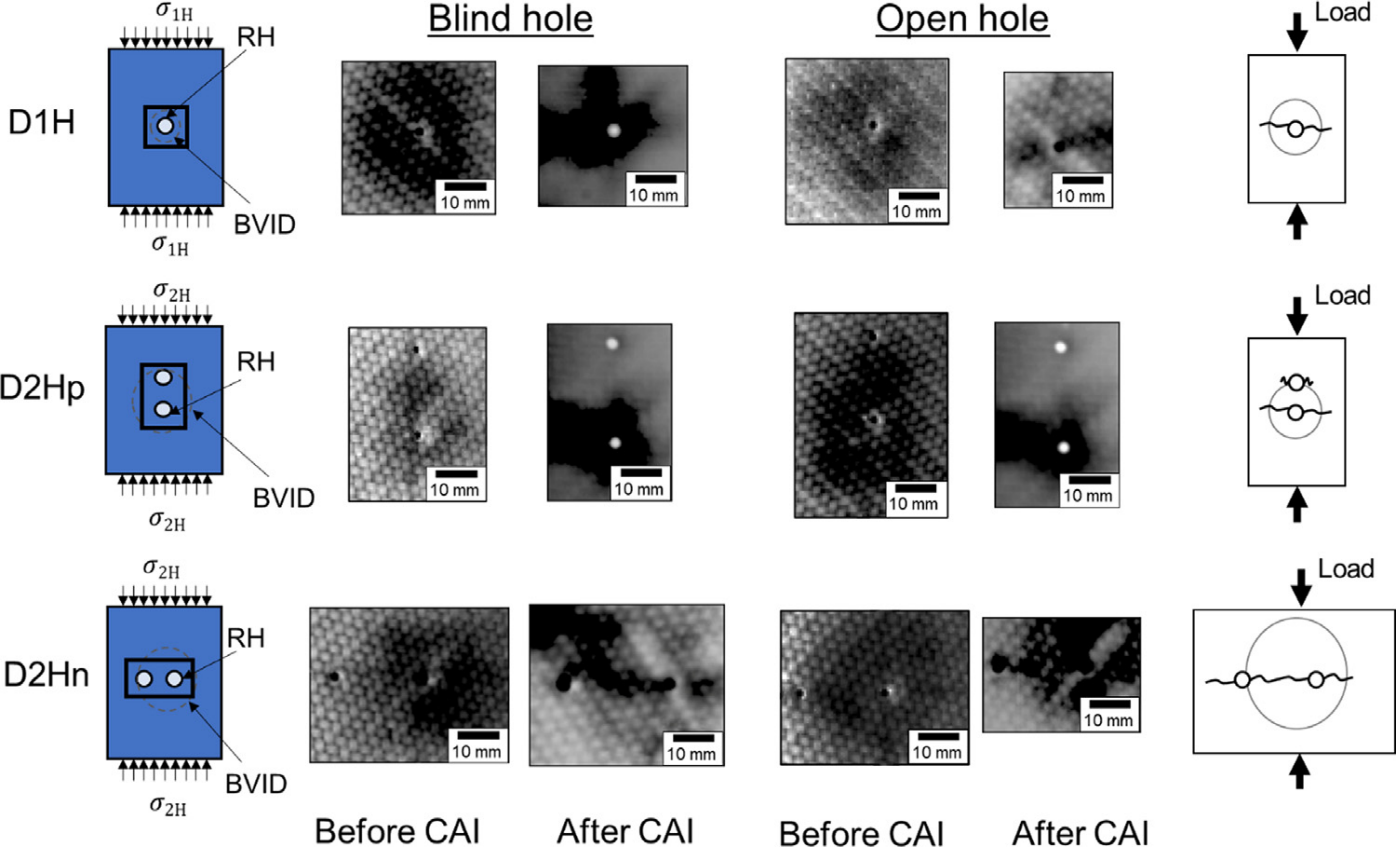
Schematics showing the hole setups and loading direction (first column) and crack propagation after test (last column). Thermograph showing structural failure around holes in BVID before and after compression after impact (CAI, i.e. in-plane compression) tests by using infrared thermography.

Fig. 1 shows the dot plot of the BVID diameter created by quasi-static indentation (QSI) in CFRP specimens. The quantitative structural data namely BVID diameter and the drilling-induced hole damage size can be found in the respective spreadsheet in the supplementary excel file (Data_Mech_Struct). Fig. 2 shows the schematic of hole setups and crack propagation around holes, and thermographs showing the typical structural failure around the blind holes and open holes in BVID before and after compression-after-impact (CAI) test. The complete dataset of the thermographs and digital images showing the structural failures can be found in the MS PowerPoint file (Images_of_hole.ppt). The video clips that shows crack propagation around the holes on the surface of the laminates during in-plane compression test was recorded, and the video clip can be found in the supplementary folder (Compression_test_clip.zip).

## 2 Experimental Design, Materials and Methods

### 2.1 Experimental design

**Fig. 3:**
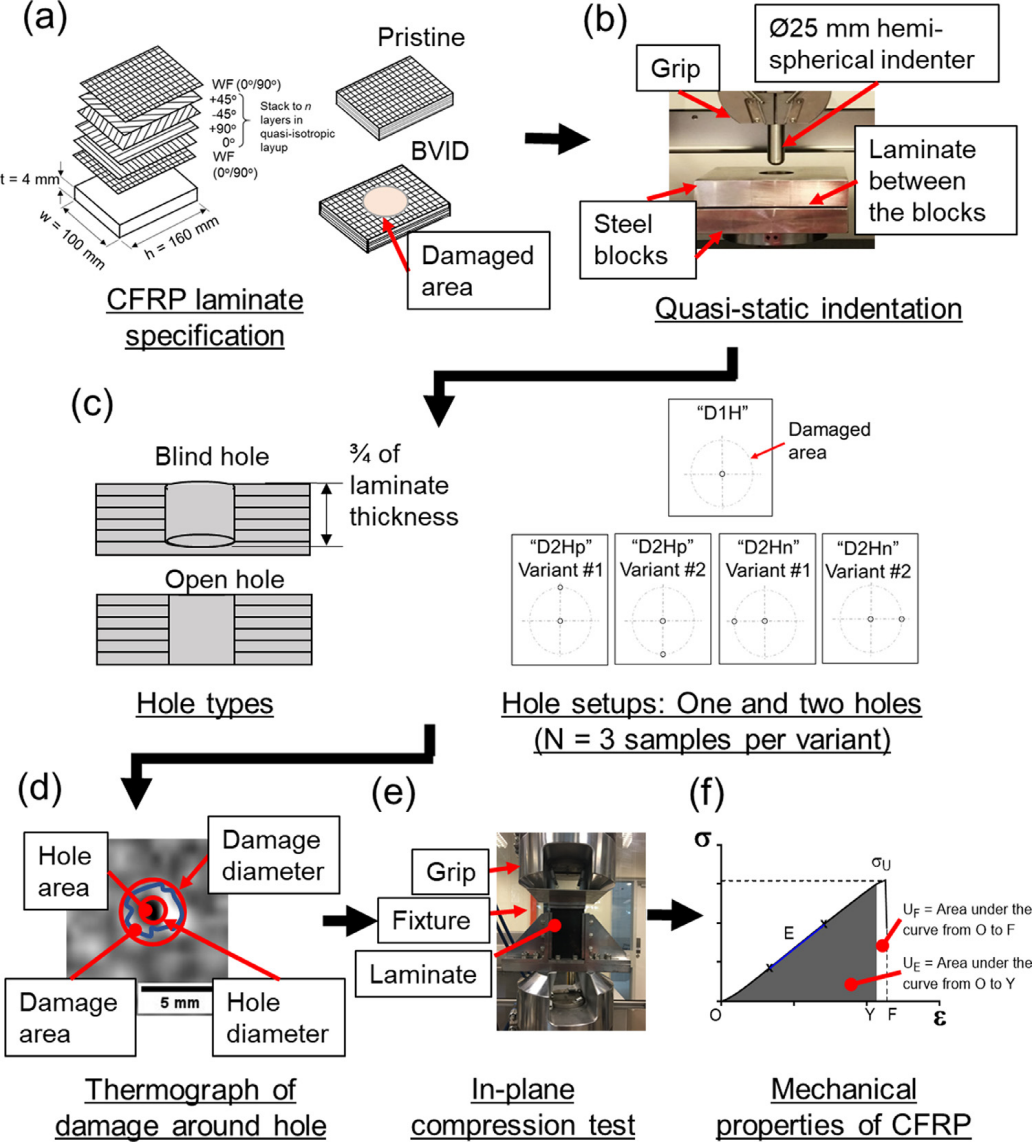
Experimental flow diagram. (a) CFRP laminate specification. (b) Quasi-static indentation. (c) Schematics of the hole types (blind and open hole) and the hole setups (single and binary-hole setups, *N* = 3 samples per variant) in BVID specimens. (d) Thermograph of the damage around the hole. (e) In-plane compression test. (f) Deriving of laminate mechanical properties from a stress-strain curve after in-plane compression test. Symbols: σ_U_ denotes compressive strength. ε denotes strain. U_E_ denotes modulus of resilience. U_F_ denotes fracture toughness. E denotes stiffness.

The data presented in Section 2 were derived from a series of experimental activities. Fig. 3 presents the diagram of the experimental workflow. The detailed information of (a) specifications of the CFRP laminate, (b) creating BVID using QSI, (c) process of drilling holes and hole setups, (d) inspection of BVID and hole damage using infrared thermography, (e) in-plane compression testing, and (f) determining the mechanical properties of the CFRP laminates were described in section 3.2 and 3.3.

### 2.2 Mechanical test data

Fig. 3(a) illustrates the schematics of the 24-ply CFRP laminates used to derive for the mechanical test data. More information regards to the specification of the 24-ply CFRP laminates could be found in another data article [3].

Table 3 shows the sample distribution for different treatments to the CFRP laminates used to derive for the mechanical test data. A total of 36 pieces of CFRP specimens were mechanically tested to rupture using in-plane compression test method. The specimens were apportioned for three treatment groups (12 specimens per group). The three-treatment group were corresponded to 1 hole, 2 holes parallel to applied load, and 2 holes normal to applied load. In each of these treatment group, the CFRP specimens were divided to 6 specimens for each type of holes namely open hole and blind hole. Of note, three out of the six specimens were subjected to BVID (BVID specimens), while the other three specimens remained as pristine (pristine specimens).

**Table 3:** Sample distribution for different treatments of the CFRP laminates used to derive for the mechanical test data.

			2 Holes parallel to		2 Holes parallel to	
	1 Hole (1H)		applied load (2Hp)		applied load (2Hn)	
	Open Hole	Blind Hole	Open Hole	Blind Hole	Open Hole	Blind Hole
Pristine specimen	3	3	3	3	3	3
Damaged specimen	3	3	3	3	3	3
Sub-total	**12**		**12**		**12**	
Total	**36**					

With regard to the BVID specimens, the CFRP laminates were subjected to impact damage using quasi-static indentation method (QSI), following the ASTM methods [4]. Fig. 3(b) shows the experimental setup of the QSI method. Of note, an impact energy of about 12 J was exerted into the CFRP samples during QSI; the imparted energy was deemed as the range of energies associated to BVID as reported in a literature [5].

Fig. 3(c) illustrates the schematics of the different hole types (blind hole and open hole) and hole setups (1H, 2Hp, and 2Hn). The holes were created by mechanical drilling using a 2-mm diameter drill bit. The different treatments to the CFRP laminates namely hole types and hole setups were related to resin-injection method used to repair BVID CFRP laminates. Several literatures reported that the blind holes [6,7] and open holes [8] were used as a preparatory steps for their repair method; but the structural effects of the CFRP laminates when such holes were introduced into the damaged site and the arrangement of the holes for the purpose of repair were not reported. With regard to the hole setup (1H, 2Hp, and 2Hn), the process of mechanical drilling of a single circular holes into the CFRP laminates removed fibres and matrix resulted in weak interfacial properties [9]. When a load was applied, the crack propagation occurs longitudinally along the weak interface; whereas at the strong interface, the cracks propagate across the fibres until the specimens failed completely [9]. Dependant on the parameters (namely the loading axis, and the specimen geometry and specification), the cracks growth may spread parallel or normal to the loading and fibre directions [9]; therefore, the two-holes setup was arranged in parallel (2Hp) or normal (2Hn) to the axis of the applied load were employed to derive for the structural and mechanical data.

Fig. 3(e) shows the experimental setup of the in-plane compression test used to derive the dataset of the mechanical properties of the CFRP samples. The in-plane compression test follows a ASTM protocol [10]. The data recorded from the mechanical test consists of the load relative to the change in displacement (ΔL). Fig. 3(f) shows a typical stress-strain (σ−ε) curve of the CFRP specimens with hole. The load and displacement data obtained was used to derive for the stress (σ) and strain (ε). The σ versus ε plots was further analysed to derive for the compressive strength (σ_U_), compressive strain at rupture (ε_U_), modulus of resilience (u_E_), fracture toughness (u_F_), and stiffness (E).

The information related to (1) QSI method used to create BVID in CFRP laminates, (2) in-plane compression test method, and (3) mechanical properties derived from the stress-strain curve can be found in another data article [3].

### 2.3 Structural data

Non-destructive testing (NDT) methods namely the infrared thermography and digital imaging was used to examine the failure structures and interaction between holes within the CFRP laminates. The infrared thermography captures the thermal variation (depicts the material inhomogeneity) on the surface of the specimens in the form of images also known as thermograph. The method used to conduct infrared thermography inspection on the CFRP specimens were similar to the procedures shown in another data article [3]. The thermograph was used to (1) quantify the damage area of the BVID, (2) determine the positions of the holes in the BVID of the specimens before mechanical drilling, (3) examine for interaction between two holes, and (4) examine for damage features around the edges of the hole [11].

For digital imaging, a digital camera (Nikon D7200) was used to capture images and videos of the failures of specimens during mechanical testing. The dataset obtained from digital imaging was used to observe the failure patterns happens on the specimens during the in-plane compression test. Videos were taken during the process of the in-plane compression test to observe how structures failed around hole or to another nearby holes [12].

## CRediT Author Statement

**Lai Wei Liang:** Conceptualization, Methodology, Data curation, Investigation, Writing – original draft. **Hamid Saeedipour:** Co-Supervision, Resource, Funding acquisition. **Goh Kheng Lim:** Supervision, Writing – reviewing and editing.

## Declaration of Competing Interest

The authors declare that they have no known competing financial interests or personal relationships which have, or could be perceived to have, influenced the work reported in this article.

## Appendix Supplementary materials

Image, application 1

## References

[1] Lai W.L., Cheah A.Y.H., Ruiz R.C.O., Lo N.G.W., Kuah K.Q.J., Saeedipour H., Goh K.L. (2017). A simple portable low-pressure healant-injection device for repairing damaged composite laminates. Int. J. Mech. Eng. Educ..

[2] Rahman M.A.A.S.B., Lai W.L., Saeedipour H., Goh K.L. (2019). Cost-effective and efficient resin-injection device for repairing damaged composites. Reinforc Plast..

[3] Lai W.L., Saeedipour H., Goh K.L. (2019). Dataset on mechanical properties of damaged fibre composite laminates with drilled vent-holes for resin-injection repair procedure. Data Brief.

[4] ASTM-D6264-17 (2017). Standard test method for measuring the damage resistance of a fiber-reinforced polymer-matrix composite to a concentrated quasi-static indentation force. ASTM Int..

[5] Sun X.C., Hallett S.R. (2017). Barely visible impact damage in scaled composite laminates: experiments and numerical simulations. Int. J. Impact Eng..

[6] Hautier M., Lévêque D., Huchette C., Olivier P. (2013). Investigation of composite repair method by liquid resin infiltration. Plast. Rubber Compos. Macromol. Eng..

[7] Thunga M., Bauer A., Obusek K., Meilunas R., Akinc M., Kessler M.R. (2014). Injection repair of carbon fiber/bismaleimide composite panels with bisphenol E cyanate ester resin. Compos. Sci. Technol..

[8] Slattery P.G., McCarthy C.T., O'Higgins R.M. (2016). Development of a novel cyanoacrylate injection repair procedure for composites. Compos. Struct..

[9] Toubal L., Karama M., Lorrain B. (2005). Stress concentration in a circular hole in composite plate. Compos. Struct..

[10] ASTM-D7137-12 (2017). Standard test method for compressive residual strength properties of damaged polymer matrix composite plates. ASTM Int..

[11] Lai W.L., Saeedipour H., Goh K.L. (2020). Mechanical properties of low-velocity impact damaged carbon fibre reinforced polymer laminates: effects of drilling holes for resin-injection repair. Compos. Struct..

[12] Soutis C., Fleck N.A., Curtis P.T. (1991). Hole-hole interaction in carbon fibre/epoxy laminates under uniaxial compression. Composites.

