# Supplementary material for: Dataset on open/blind hole-hole interaction in barely visible impact damaged composite laminates
Source: Data Brief. 2020 Dec 1;34:106607. doi: 10.1016/j.dib.2020.106607 (PMC7744944; doi:10.1016/j.dib.2020.106607)
Supplement: Supplementary file 1 [file mmc1.zip › Images_of_hole.pptx]

## Slide 1
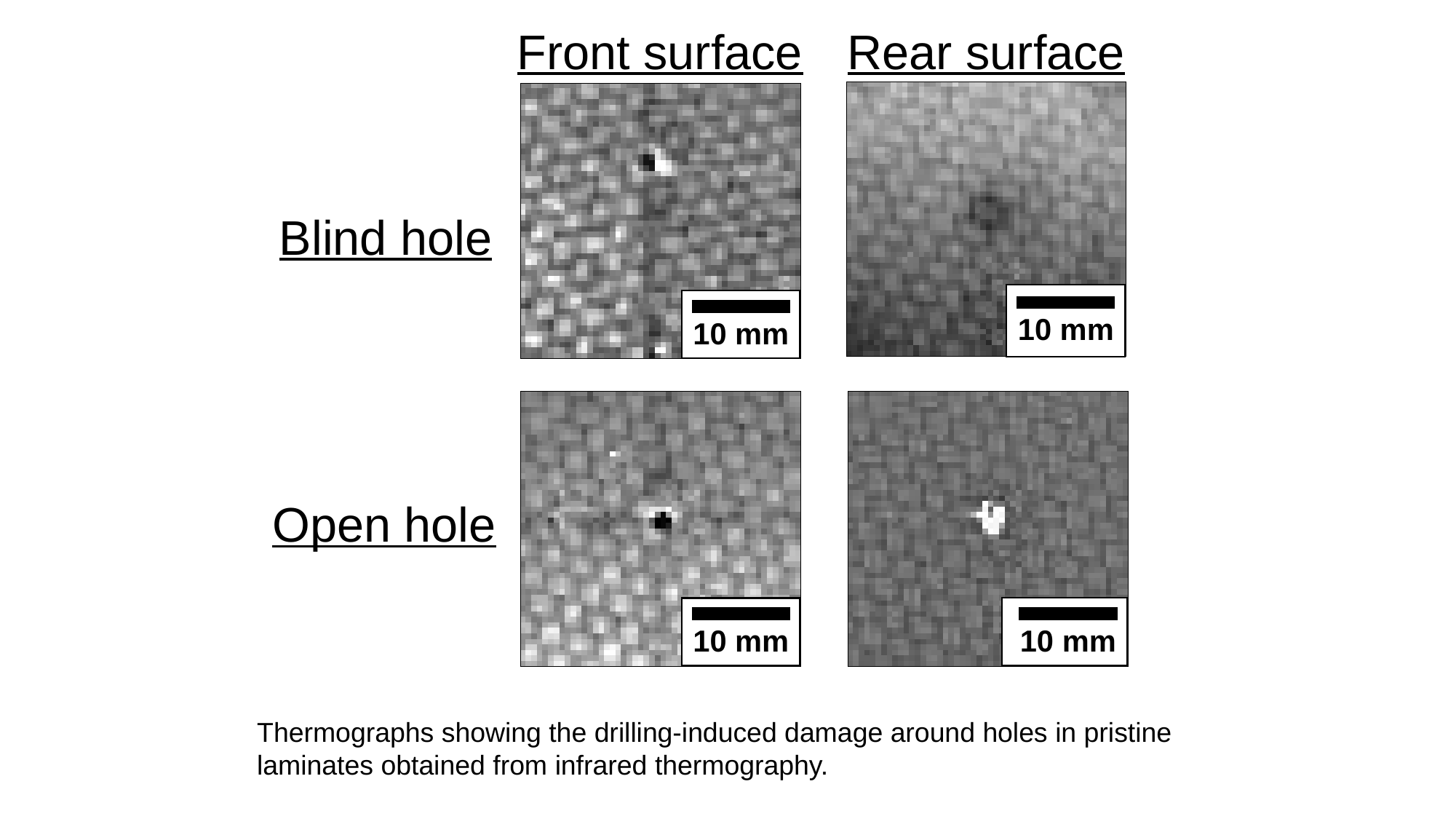

Front surface
Rear surface
10 mm
10 mm
Blind hole
10 mm
10 mm
Open hole
Thermographs showing the drilling-induced damage around holes in pristine laminates obtained from infrared thermography.

## Slide 2
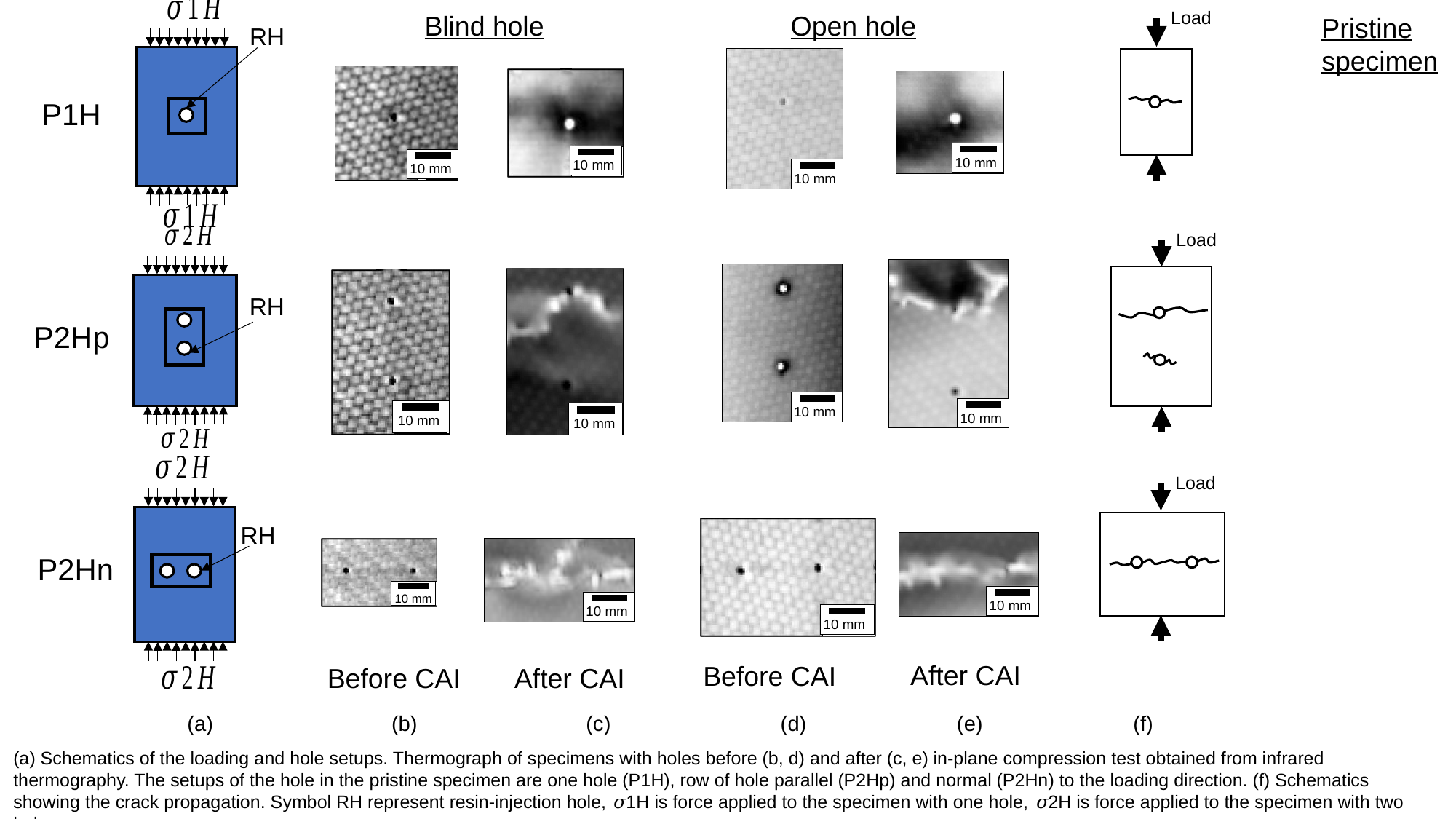

Load
Blind hole
Open hole
RH
10 mm
10 mm
10 mm
10 mm
P1H
Load
10 mm
10 mm
10 mm
10 mm
RH
P2Hp
Load
RH
10 mm
10 mm
10 mm
10 mm
P2Hn
After CAI
Before CAI
After CAI
Before CAI
(a)
(b)
(c)
(d)
(e)
(f)
Pristine specimen
(a) Schematics of the loading and hole setups. Thermograph of specimens with holes before (b, d) and after (c, e) in-plane compression test obtained from infrared thermography. The setups of the hole in the pristine specimen are one hole (P1H), row of hole parallel (P2Hp) and normal (P2Hn) to the loading direction. (f) Schematics showing the crack propagation. Symbol RH represent resin-injection hole, 𝜎1H is force applied to the specimen with one hole, 𝜎2H is force applied to the specimen with two holes.

## Slide 3
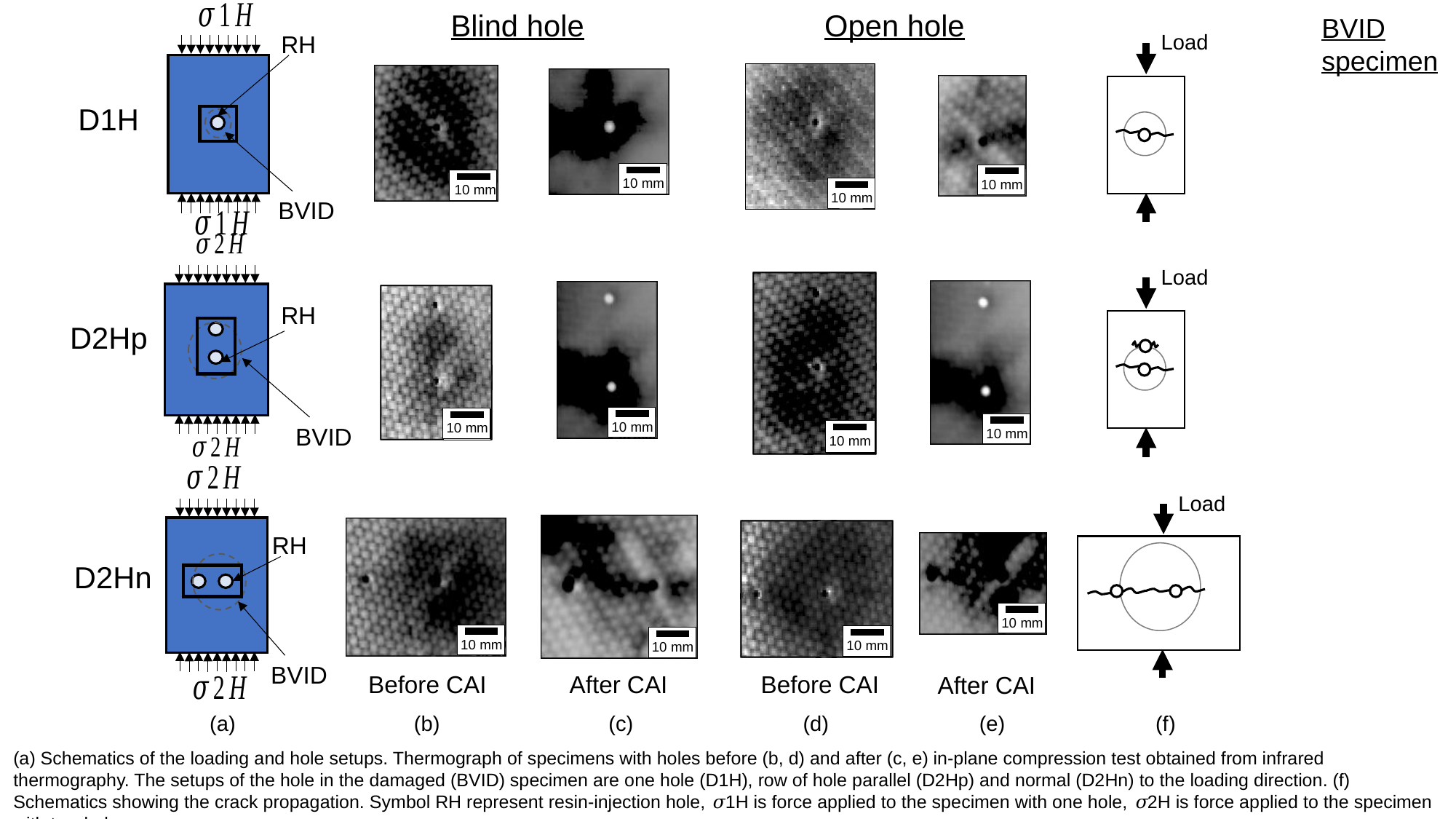

Blind hole
Open hole
RH
Load
10 mm
10 mm
10 mm
10 mm
D1H
BVID
Load
10 mm
10 mm
10 mm
10 mm
RH
D2Hp
BVID
Load
10 mm
10 mm
10 mm
RH
10 mm
D2Hn
BVID
Before CAI
After CAI
Before CAI
After CAI
(a)
(b)
(c)
(d)
(e)
(f)
BVID specimen
(a) Schematics of the loading and hole setups. Thermograph of specimens with holes before (b, d) and after (c, e) in-plane compression test obtained from infrared thermography. The setups of the hole in the damaged (BVID) specimen are one hole (D1H), row of hole parallel (D2Hp) and normal (D2Hn) to the loading direction. (f) Schematics showing the crack propagation. Symbol RH represent resin-injection hole, 𝜎1H is force applied to the specimen with one hole, 𝜎2H is force applied to the specimen with two holes.

## Slide 4
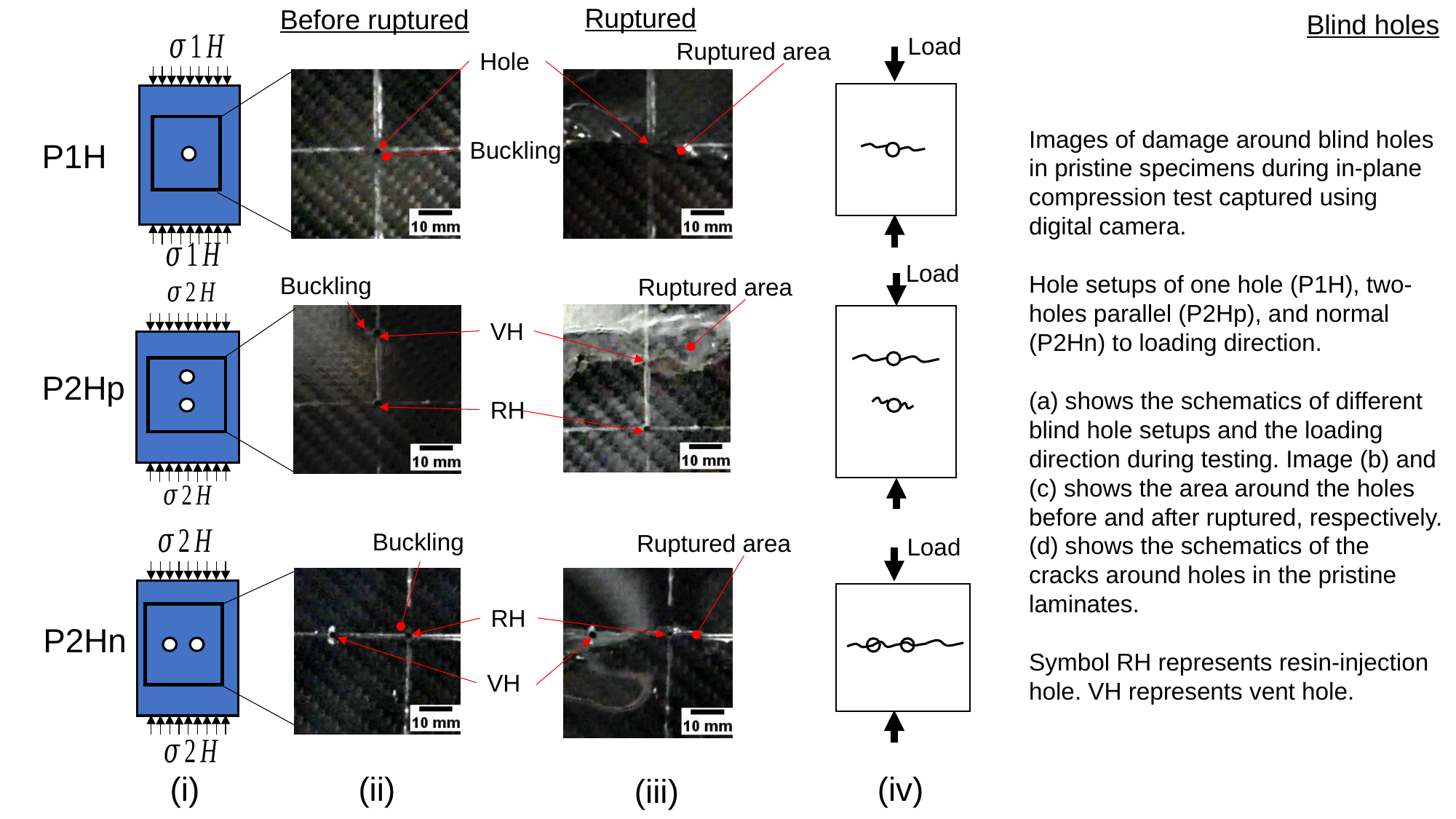

Ruptured
Before ruptured
Load
Ruptured area
Hole
Buckling
P1H
Load
Buckling
Ruptured area
VH
P2Hp
RH
Buckling
Ruptured area
Load
RH
P2Hn
VH
(i)
(ii)
(iv)
(iii)
Blind holes
Images of damage around blind holes in pristine specimens during in-plane compression test captured using digital camera.
Hole setups of one hole (P1H), two-holes parallel (P2Hp), and normal (P2Hn) to loading direction.
(a) shows the schematics of different blind hole setups and the loading direction during testing. Image (b) and (c) shows the area around the holes before and after ruptured, respectively. (d) shows the schematics of the cracks around holes in the pristine laminates.
Symbol RH represents resin-injection hole. VH represents vent hole.

## Slide 5
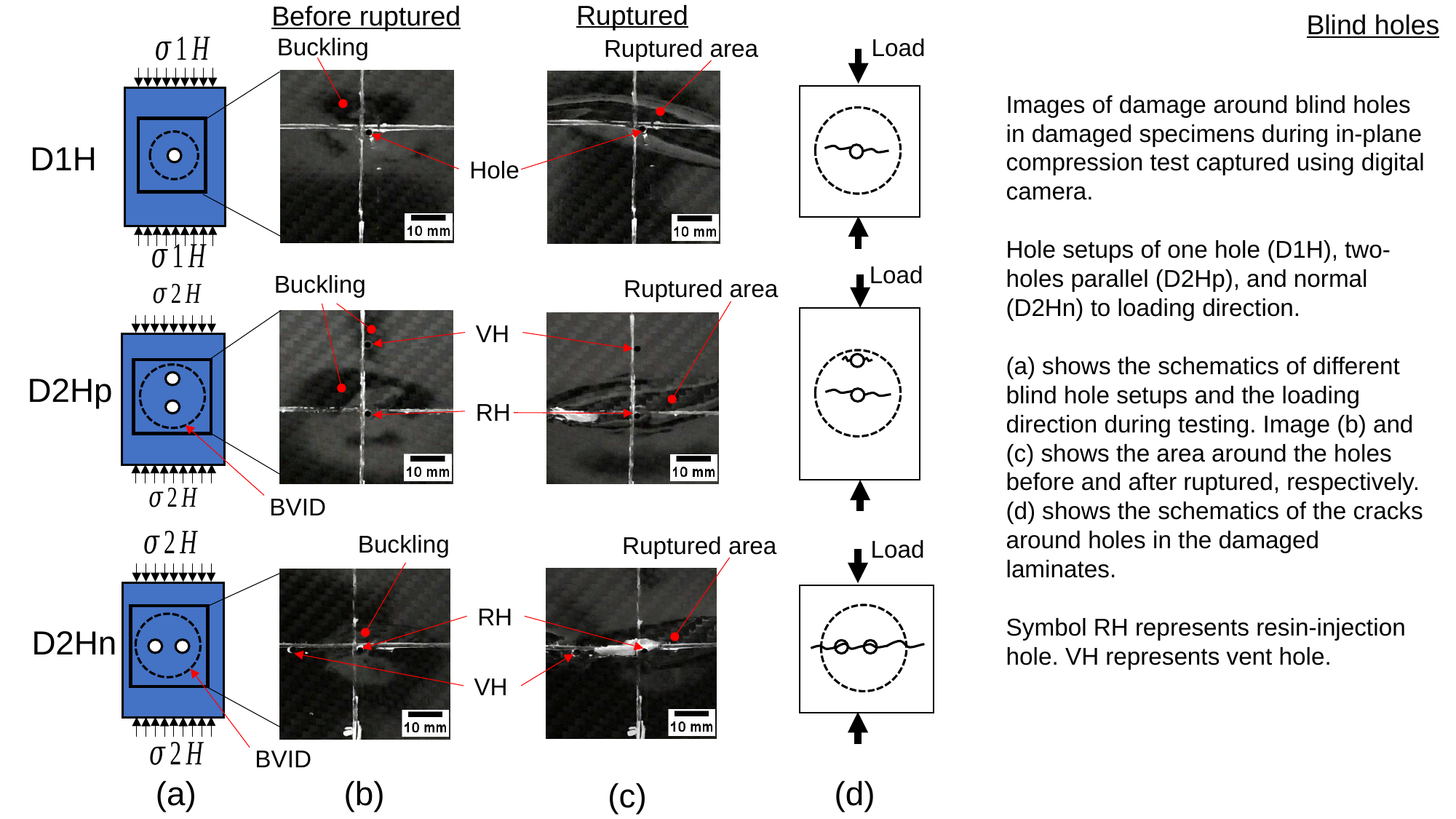

Ruptured
Before ruptured
Buckling
Load
Ruptured area
D1H
Hole
Load
Buckling
Ruptured area
VH
D2Hp
RH
BVID
Buckling
Ruptured area
Load
RH
D2Hn
VH
BVID
(a)
(b)
(d)
(c)
Blind holes
Images of damage around blind holes in damaged specimens during in-plane compression test captured using digital camera.
Hole setups of one hole (D1H), two-holes parallel (D2Hp), and normal (D2Hn) to loading direction.
(a) shows the schematics of different blind hole setups and the loading direction during testing. Image (b) and (c) shows the area around the holes before and after ruptured, respectively. (d) shows the schematics of the cracks around holes in the damaged laminates.
Symbol RH represents resin-injection hole. VH represents vent hole.

## Slide 6
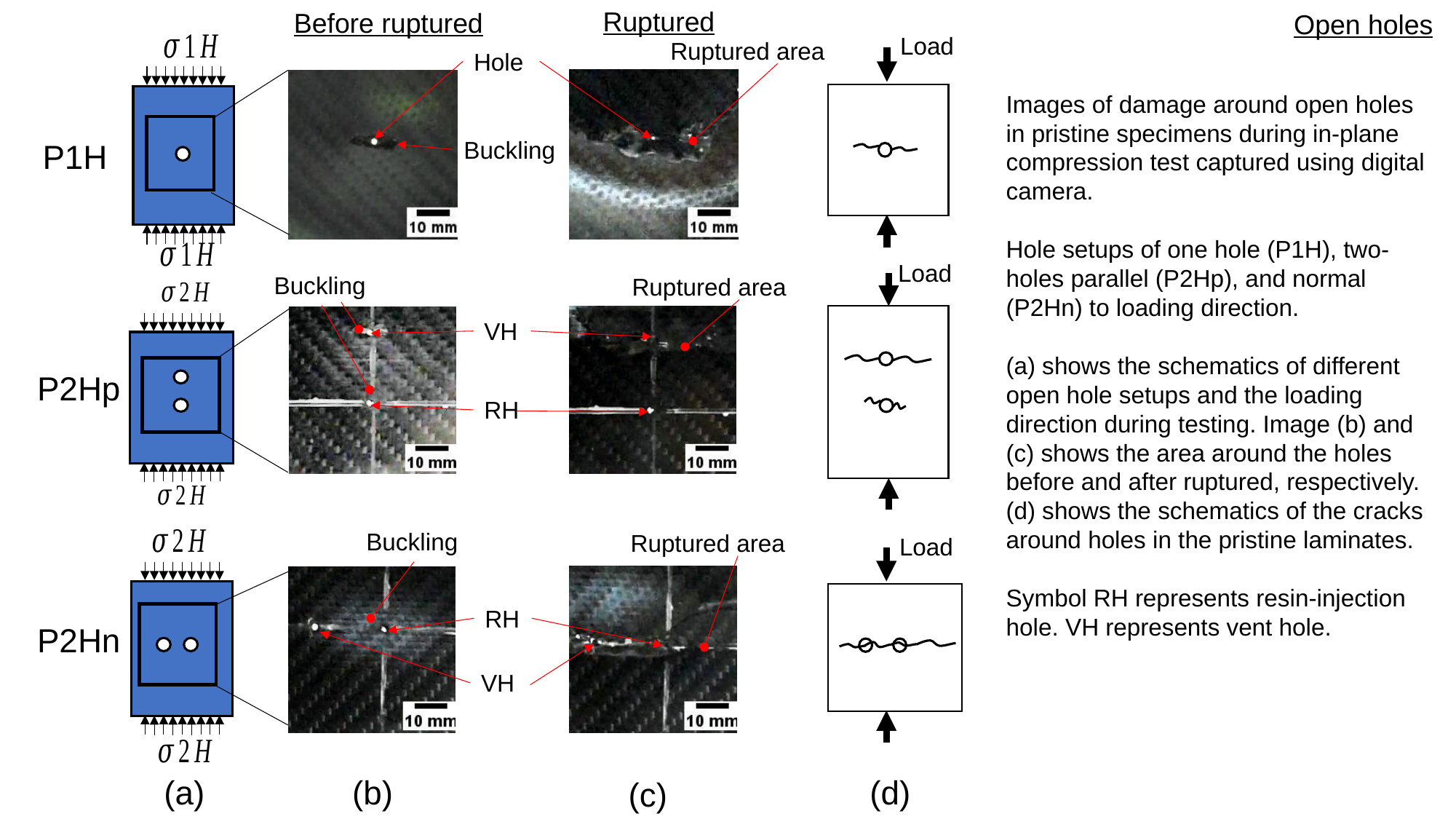

Ruptured
Before ruptured
Load
Ruptured area
Hole
Buckling
P1H
Load
Buckling
Ruptured area
VH
P2Hp
RH
Buckling
Ruptured area
Load
RH
P2Hn
VH
(a)
(b)
(d)
(c)
Open holes
Images of damage around open holes in pristine specimens during in-plane compression test captured using digital camera.
Hole setups of one hole (P1H), two-holes parallel (P2Hp), and normal (P2Hn) to loading direction.
(a) shows the schematics of different open hole setups and the loading direction during testing. Image (b) and (c) shows the area around the holes before and after ruptured, respectively. (d) shows the schematics of the cracks around holes in the pristine laminates.
Symbol RH represents resin-injection hole. VH represents vent hole.

## Slide 7
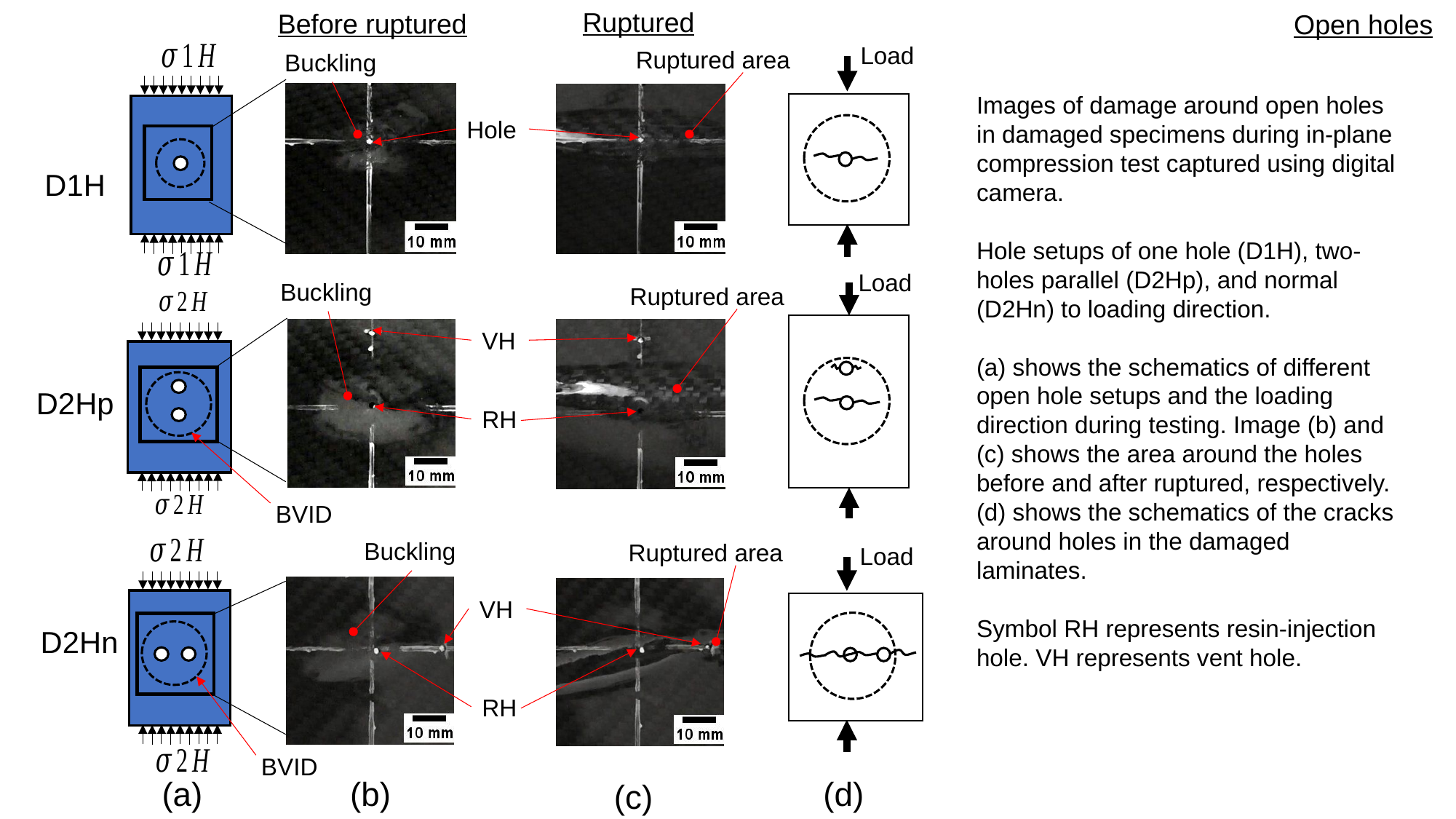

Ruptured
Before ruptured
Load
Ruptured area
Buckling
Hole
D1H
Load
Buckling
Ruptured area
VH
D2Hp
RH
BVID
Buckling
Ruptured area
Load
VH
D2Hn
RH
BVID
(a)
(b)
(d)
(c)
Open holes
Images of damage around open holes in damaged specimens during in-plane compression test captured using digital camera.
Hole setups of one hole (D1H), two-holes parallel (D2Hp), and normal (D2Hn) to loading direction.
(a) shows the schematics of different open hole setups and the loading direction during testing. Image (b) and (c) shows the area around the holes before and after ruptured, respectively. (d) shows the schematics of the cracks around holes in the damaged laminates.
Symbol RH represents resin-injection hole. VH represents vent hole.
